# Estimated pulse wave velocity as a predictor of all-cause and cardiovascular mortality in patients with hypertension in China: a prospective cohort study

**DOI:** 10.3389/fcvm.2024.1365344

**Published:** 2024-04-29

**Authors:** Yumeng Shi, Chao Yu, Wei Zhou, Tao Wang, Lingjuan Zhu, Huihui Bao, Ping Li, Xiaoshu Cheng

**Affiliations:** ^1^ Department of Cardiovascular Medicine, the Second Affiliated Hospital, Jiangxi Medical College, Nanchang University, Nanchang, Jiangxi, China; ^2^ Jiangxi Provincial Cardiovascular Disease Clinical Medical Research Center, Nanchang, Jiangxi, China; ^3^ Jiangxi Sub-Center of National Clinical Research Center for Cardiovascular Diseases, Nanchang, Jiangxi, China; ^4^ Center for Prevention and Treatment of Cardiovascular Diseases, the Second Affiliated Hospital, Jiangxi Medical College, Nanchang University, Nanchang, Jiangxi, China

**Keywords:** estimating pulse wave velocity, brachial-ankle pulse wave velocity, all-cause mortality, cardiovascular mortality, hypertensive

## Abstract

**Background:**

Whether the estimated pulse wave velocity (ePWV) in Chinese patients with hypertension can serve as an independent predictor of cardiovascular and all-cause mortality remains unknown. Therefore, this study investigated the associations between ePWV and cardiovascular and all-cause mortalities and explored potential effect modifiers influencing these relationships. Finally, we compared the ePWV with the brachial-ankle pulse wave velocity (baPWV) to determine which parameter better predicts mortality.

**Methods:**

The population of this longitudinal cohort study was selected from the China H-type Hypertension Registry Study. The exposure and outcome variables were ePWV and all-cause and cardiovascular mortalities, respectively. The Cox proportional hazard regression model was applied to assess the associations between ePWV and all-cause and cardiovascular mortalities. The performances of ePWV and baPWV in predicting death were compared using the receiver operating characteristic (ROC) curve area, net reclassification improvement index (NRI), and integrated discrimination improvement index (IDI).

**Results:**

This prospective study enrolled 14,232 patients with hypertension. Following an average follow-up of 48 months, 806 individuals succumbed to all-cause mortality, with 397 cases specifically attributed to cardiovascular diseases. The Cox proportional regression analysis revealed a significant association between a 1 m/s increase in ePWV and a 37% higher risk of all-cause mortality (hazard ratio [HR]: 1.37, 95% confidence interval [CI]: 1.31–1.43) as well as a 52% higher risk of cardiovascular mortality (HR: 1.52, 95% CI: 1.43–1.62) in the fully adjusted model. The findings for ePWV according to quartile demonstrated hazard ratios for all-cause mortality for Q2 (10.25 < ePWV < 11.32), Q3 (11.32 < ePWV < 12.40), and Q4 (ePWV ≥ 12.40) of 1.50 (HR: 1.50, 95% CI: 1.07–2.10), 2.34 (HR: 2.34, 95% CI: 1.73–3.18), and 4.09 (HR: 4.09, 95% CI: 3.05–5.49), respectively, compared with Q1 (ePWV < 10.25). The risk of cardiovascular death also increased in proportion to the rise in ePWV. The results of the area under the ROC curve, NRI, and IDI all indicated that ePWV outperformed baPWV in predicting mortality. The results of the subgroup analysis demonstrated that body mass index (BMI) and hypoglycemic drug use modified the association between ePWV and mortality.

**Conclusions:**

The performance of ePWV in predicting all-cause and cardiovascular mortalities was superior to that of baPWV alone. Patients who were overweight or obese with higher ePWV values exhibited a significantly increased risk of all-cause death. The correlation between elevated ePWV and the risk of cardiovascular death was more pronounced in patients who had not received hypoglycemic drugs.

## Introduction

## Background

The Global Report on the Burden of Cardiovascular Diseases has reported a progressive escalation of the global burden of cardiovascular disease between 1999 and 2019. Cardiovascular disease affects 523 million individuals worldwide, with 18.6 million deaths (1).The close association between aging, cardiovascular disease, and mortality cannot be disregarded as a significant risk factor for cardiovascular disease. Aging exacerbates susceptibility to cardiovascular diseases, thereby increasing global mortality rates (2, 3). With recent advances in economic development and the escalating challenges posed by population aging, the burden of cardiovascular disease has increased significantly in China. Age-related cardiovascular diseases have emerged as the primary cause of mortality in both urban and rural populations. Progressive stiffening of blood vessel walls is an inherent physiological characteristic associated with vascular aging, which is further exacerbated by age progression and chronic non-communicable diseases. Arterial stiffness is also a crucial indicator marking the process of aging and plays a pivotal role in assessing vascular health (4). Arterial stiffness has also been linked to the development of cardiovascular diseases, cerebrovascular diseases, and diabetes, with increased stiffness associated with significantly elevated risks for these conditions (5–8). Moreover, chronic hypertension can directly induce physical damage to the vascular wall, leading to reduced arterial wall elasticity and increased vascular stiffness. This increase subsequently elevates the susceptibility to hypertension, establishing a reciprocal causal relationship between these two factors (6). Thus, the measurement of arterial stiffness has gained increasing recognition in recent years for both assessing disease risk but also as a supplementary intervention to promote stiffness reversal. Therefore, regular monitoring of arterial stiffness and the early implementation of intervention measures in patients with hypertension are of paramount significance in reducing their risks of cardiovascular events and mortality.

The main indicators for detecting arterial stiffness differ according to region, with carotid-femoral pulse wave velocity (cfPWV) used in Europe (9, 10) and America (11) and brachial-ankle PWV (baPWV) in Asia (12, 13). Previous research has demonstrated the strong correlation between these measures (14). Moreover, the American Heart Association has endorsed baPWV as a widely employed approach for assessing arterial stiffness in clinical settings among Asian populations (Class I, level of evidence B) (15). Although cfPWV and baPWV are standardized, simple, and non-invasive, their effective implementation necessitates specialized equipment and expertise. Consequently, their widespread application in clinical practice is often limited to medical institutions of Grade II or higher. This limitation poses challenges in conducting such assessments, particularly in ordinary community healthcare units, rural areas with limited medical resources, and during special epidemic periods. Therefore, researchers have developed and verified the estimated PWV (ePWV) as a measure to replace direct measures of PWV (16). The ePWV only requires age and average blood pressure to easily obtain results. Recently, ePWV has been shown to be an independent predictor of cardiovascular disease events and survival status (17–24). However, whether ePWV can independently predict cardiovascular and all-cause mortality in Chinese patients with hypertension and whether it is superior to baPWV in predicting mortality remains unclear. Further research and verification are warranted to evaluate the predictive value and potential applications of ePWV in this population.

Therefore, the present study assessed the predictive capability of ePWV for cardiovascular and all-cause mortalities among patients with hypertension and compared its performance with that of baPWV. Additionally, we investigated potential effect modifiers between ePWV and cardiovascular and all-cause mortalities.

## Methods

### Study population

The data used in this study were obtained from the China H-type Hypertension Registry Study (registration number: ChiCTR1800017274;20/07/2018), an ongoing real-world prospective cohort study in Wuyuan, China, which began in March 2018. This registration study has been previously described in detail (25). Briefly, patients >18 years of age are eligible for study participation study if they (1) have been diagnosed with hypertension (systolic blood pressure [SBP] ≥ 140 mmHg or diastolic blood pressure [DBP] ≥ 90 mmHg) or are taking antihypertensive drugs during the screening phase, and (2) provide informed written consent. The exclusion criteria are as follows: (1) mental or nervous system dysfunction or inability to express their will, (2) inability to complete the follow-up as required or planning to migrate to the field soon, and (3) patients considered by the investigator to be unsuitable for inclusion in the group or for long-term follow-up. The study protocol was approved by the Ethics Committees of the Institute of Biology at Anhui Medical University (No. CH1059) and the Second Affiliated Hospital of Nanchang University (No. 2018019).

**Figure 1:**
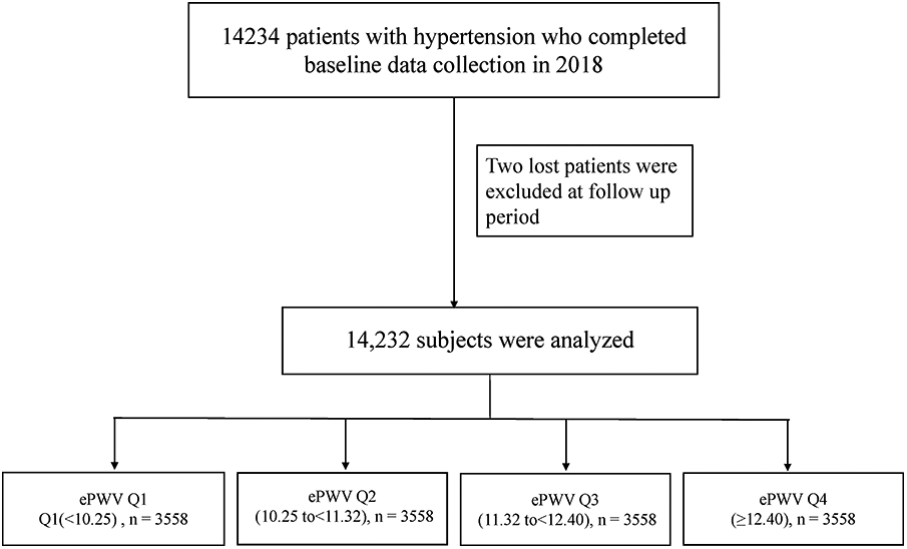
Flow chart of study participants.

At baseline, in March 2018, we examined the study population of 14,234 patients with hypertension. All patients were followed up until August 2022. After excluding two participants lost to follow-up, the final analysis included 14,232 patients with hypertension (Figure 1).

### Data collection

All participants underwent baseline and follow-up health assessments using a structured modified screening questionnaire to determine their demographic characteristics, including age, sex, physical activity, smoking history, drinking history, medical history, and medications. The anthropometric indices included height, weight, and waist circumference. Body mass index (BMI) was defined as body weight/height^2^ (kg/m^2^). Trained medical staff assessed blood pressure (BP) to limit interobserver variability. After the participants rested for 5 min, seated BP and heart rate were measured using an electronic sphygmomanometer (Omron; Dalian, China) following the standard method and using appropriately sized cuffs. Three measurements on the right arm were performed at 1-min intervals between successive readings, and the mean value was calculated. Alcohol consumption was defined as drinking an average of two or more times per week over a year. Drinking habits were categorized as occasional (drinking alcohol monthly or less) or regular drinking (drinking alcohol at least twice per month). Current smoking was defined as smoking ≥1 cigarette per day for ≥1 year or a cumulative smoking amount of ≥360 cigarettes per year.

Blood samples were collected after the participants had fasted for 8–12 h. The samples were quickly processed to obtain serum and stored at −80°C until they were sent for analysis (Biaojia Biotechnology, Shenzhen, Guangdong Province, China). Automatic clinical analyzers (Beckman Coulter, USA) were used to measure all biochemical parameters including fasting plasma glucose (FPG), homocysteine (Hcy), triglyceride (TG), total cholesterol (TC), high-density lipoprotein cholesterol (HDL-C), low-density lipoprotein cholesterol (LDL-C), serum albumin, and serum uric acid levels. The glomerular filtration rate (eGFR) was estimated using the Chronic Kidney Disease Epidemiology Collaboration (CKD-EPI) equation (26). Diabetes mellitus was defined as a self-reported physician diagnosis of diabetes, FPG concentration ≥7.0 mmol/L, or use of hypoglycemic drugs. Medical history of coronary heart disease (CHD) and stroke were self-reported by the participants primarily through the administration of questionnaires. Each participant was queried regarding the presence of corresponding symptoms, type of treatment received, and availability of pertinent medical records (including discharge summaries, biochemical examination data, and imaging data) during episodes of CHD or stroke. Atrial fibrillation (AF) was defined as the presence of AF on a standard 12-lead electrocardiogram or a documented history of AF.

### Outcome assessments

The primary and secondary outcome measures of this longitudinal cohort study were all-cause and cardiovascular mortalities, respectively. Data on deaths were collected by three means from August 31, 2018–August 31, 2022. First, a professionally trained Clinical Research Coordinator (CRC) conducted telephone follow-ups and household surveys. Second, community hospitals in the affiliated jurisdictions of Wuyuan County provided death certificates. Third, we extracted the death information of the subjects from the Wuyuan County Medical Security Bureau, reviewed the endpoint events collected as described above by the endpoint committee from each participant's inpatient and outpatient medical records, and determined the type of endpoint event. The all-cause deaths included those attributed to (1) cardiovascular diseases, (2) malignant tumors, and (3) other causes (1). Cardiovascular disease was defined as a patient's medical history indicating a diagnosis by a class II hospital or above, including cardiovascular disease (myocardial infarction and heart failure) and stroke (cerebral infarction, cerebral hemorrhage, and subarachnoid hemorrhage) (27). Cardiovascular death was defined as death due to sudden cardiac death, myocardial infarction, heart failure, stroke, invasive cardiovascular surgery, or cardiovascular hemorrhage during the follow-up period (28).

### Exposure variables

The exposure variable was ePWV. We used a formula derived from the Reference Values for Arterial Stiffness' Collaboration (29), as described by Greve et al. (16) ePWV was calculated using age (years) and mean blood pressure (MBP, mmHg) as follows: ePWV = 9.587−0.402 × age + 4.560 × 10^−3^ × age^2^−2.621 × 10^−5^ × age^2^ × MBP + 3.176 × 10^−3^ × age × MBP−1.832 × 10^−2^ × MBP. MBP was calculated as DBP + 0.4 (SBP–DBP). In this study, 0.4 was used to calculate MBP instead of 0.33 as a coefficient of 0.4 is more consistent with the change in heart rate contour with age and its relationship with target organ damage (30, 31). In addition, baPWV was detected in selected populations in this study. baPWV (cm/s) was automatically measured simultaneously using an automatic waveform analyzer (BP-203RPE III device; Omron Health Care, Kyoto, Japan) with the participants in the supine position after resting for >10 min.

### Statistical analysis

All data in this study were analyzed using Empower (R; www.empowerstats.com; X&Y Solutions, Inc, Boston, MA, USA) and the R language (http://www.R-project.org, The R Foundation). For all analyses, two-tailed *P* < 0.05 was considered statistically significant.

We used means with standard deviations and medians with interquartile ranges to analyze continuous variables with normal and non-normal distributions, respectively. Categorical variables are presented as frequencies and percentages. The study population was divided according to ePWV quartiles. Analysis of variance (ANOVA) and the chi-square test were used to explore differences in quartile characteristics at the ePWV level for continuous and categorical variables, respectively. The proportionality of the hazards assumption was assessed using the Schoenfeld residuals technique. If the proportional hazard assumption was satisfied (*p* = 0.29 for testing departures from proportionality), Cox proportional hazard models were used to estimate the associations of ePWV and baPWV with mortality after adjusting for study covariates. We constructed three models: Model 1 was unadjusted. Model 2 was adjusted for sex, BMI, and heart rate. Model 3 was additionally adjusted for diabetes; stroke; CHD; AF; smoking status; drinking status; Hcy, FPG, TC, TG, uric acid, HDL-c, LDL-c, serum albumin, and eGFR levels; and antihypertensive, hypoglycemic, lipid-lowering, and antiplatelet drug use. The dose-response relationship between ePWV and cardiovascular and all-cause mortality was plotted using a generalized additive model and smooth-fitting curves. Since we tested baPWV levels in 5,232 patients at baseline to ensure that the data were unbiased, we compared the predictive power of ePWV and baPWV in these populations. The predictive values of ePWV and baPWV for cardiovascular mortality and all-cause mortality were compared using the area under the curve (AUC) of the receiver operating characteristic (ROC), net reclassification improvement (NRI), and integrated discrimination improvement (IDI). In the Cox proportional risk regression model, the risk duration was defined as the time from study initiation to cardiovascular or all-cause deaths. The Kaplan–Meier curve (log-rank test) was used to evaluate cumulative cardiovascular and all-cause mortalities according to ePWV quartile. In addition, we performed a stratified analysis interaction test and evaluated the presence of modifying factors that altered the effect of the ePWV on all-cause and cardiovascular mortalities. The robustness of the results was evaluated using several sensitivity analyses.

## Results

Among a total of 14,232 hypertensive patients enrolled in this longitudinal cohort study, 47.22% were male, with a mean age of 63.81 ± 9.36 years. The mean baseline ePWV value was 11.39 m/s. During the average follow-up period of 48 months, 806 participants died, including 397 due to CVD. The baseline characteristics of the participants according to ePWV quartile are shown in Table 1. The participants with the highest ePWV tended to be male, older, and less likely to drink than those with the lowest ePWV. Compared with patients with the lowest ePWV, those with the highest ePWV were significantly associated with the following parameters: lower BMI; higher SBP, DBP, heart rate, and Hcy; lower FPG; higher TC; lower TG; higher HDL-C; lower LDL-C; higher uric acid; lower serum albumin and eGFR; lower prevalence of diabetes; higher prevalence of CHD and AF; and less use of antihypertensive, antihypertensive, and lipid-lowering drugs. Moreover, current smoking status, prevalence of stroke, and antiplatelet drug use did not differ significantly among the participants in the four groups.

**Table 1:** Baseline characteristics of study participants according to ePWV.

Variable^a^	Total	ePWV,m/s				*P* value
		Quartile 1	Quartile 2	Quartile 3	Quartile 4	
		(<10.25)	(10.25–<11.32)	(11.32–<12.40)	(≥12.40)	
Participants	14,232	3,558	3,558	3,558	3,558	
Males, N	6,720 (47.22%)	1,719 (48.31%)	1,591 (44.72%)	1,673 (47.02%)	1,737 (48.82%)	0.003
Age,year	63.81 ± 9.36	53.49 ± 6.49	61.58 ± 5.51	66.58 ± 4.86	73.59 ± 6.31	<0.001
BMI, kg/m^2^	23.61 ± 3.74	24.63 ± 3.48	23.94 ± 3.50	23.36 ± 3.44	22.50 ± 4.18	<0.001
Current smoking, *N* (%)	3,662 (25.74%)	891 (25.05%)	932 (26.21%)	942 (26.48%)	897 (25.21%)	0.402
Current drinking, *N* (%)	3,067 (21.56%)	762 (21.42%)	801 (22.53%)	803 (22.58%)	701 (19.70%)	0.008
SBP, mmHg	148 ± 18	135 ± 13	145 ± 14	151 ± 14	162 ± 19	<0.001
DBP, mmHg	89 ± 11	88 ± 10	88 ± 11	89 ± 10	90 ± 12	<0.001
heart rate, bpm	77 ± 14	77 ± 13	76 ± 13	76 ± 15	78 ± 16	<0.001
baPWV,m/s	18.57 ± 4.21	15.66 ± 2.47	17.56 ± 2.87	18.97 ± 3.47	21.68 ± 4.87	<0.001
Hcy, *μ*mol/L	15.01 (12.47-19.13)	13.73 (11.65-17.12)	14.32 (12.21-17.90)	15.29 (12.79-19.19)	17.06 (13.75-21.62)	<0.001
FPG, mmol/L	6.18 ± 1.61	6.18 ± 1.65	6.25 ± 1.73	6.18 ± 1.53	6.12 ± 1.51	0.012
TC, mmol/L	5.16 ± 1.12	5.09 ± 1.13	5.20 ± 1.11	5.18 ± 1.10	5.16 ± 1.12	<0.001
TG, mmol/L	1.47 (1.04-2.16)	1.66 (1.16-2.43)	1.53 (1.08-2.24)	1.43 (1.02-2.06)	1.31 (0.95-1.86)	<0.001
HDL-C, mmol/L	1.51 (1.26-1.81)	1.44 (1.20-1.72)	1.51 (1.27-1.80)	1.54 (1.29-1.84)	1.57 (1.30-1.89)	<0.001
LDL-C, mmol/L	2.98 ± 0.81	3.00 ± 0.83	3.02 ± 0.81	2.99 ± 0.81	2.93 ± 0.81	<0.001
Uric acid, mmol/L	418.87 ± 120.58	418.06 ± 122.98	415.14 ± 121.65	416.54 ± 119.75	425.74 ± 117.64	<0.001
Serum albumin, mmol/L	46.63 ± 4.05	47.41 ± 3.99	46.96 ± 3.94	46.47 ± 3.94	45.69 ± 4.14	<0.001
eGFR, ml/min/1.73 m^2^	88.18 ± 20.22	97.81 ± 18.22	91.65 ± 17.72	86.12 ± 18.79	77.12 ± 20.15	<0.001
Diabetes^b^	2,618 (18.40%)	683 (19.20%)	695 (19.53%)	665 (18.69%)	575 (16.16%)	<0.001
Stroke	982 (6.90%)	242 (6.80%)	242 (6.80%)	243 (6.83%)	255 (7.17%)	0.912
CHD	729 (5.12%)	110 (3.09%)	152 (4.27%)	228 (6.41%)	239 (6.72%)	<0.001
AF	388 (2.73%)	56 (1.57%)	59 (1.66%)	95 (2.67%)	178 (5.00%)	<0.001
Antihypertensive drugs	9,226 (64.84%)	2,365 (66.49%)	2,326 (65.41%)	2,309 (64.91%)	2,226 (62.56%)	0.005
Hypoglycemic drugs	755 (5.30%)	210 (5.90%)	222 (6.24%)	181 (5.09%)	142 (3.99%)	<0.001
Lipid-lowering drugs	506 (3.56%)	145 (4.08%)	133 (3.74%)	126 (3.54%)	102 (2.87%)	0.045
Antiplatelet drugs	546 (3.84%)	132 (3.71%)	120 (3.37%)	140 (3.93%)	154 (4.33%)	0.199

ePWV, estimated pulse wave velocity; BMI, body mass index; SBP, systolic blood pressure; DBP, diastolic blood pressure; FPG: fasting plasma glucose; TC total cholesterol; TG triglyceride; HDL-C, High-density lipoprotein cholesterol; LDL-C, Low-density lipoprotein cholesterol; SUA, serum uric acid; eGFR, estimated glomerular filtration rate; CHD, coronary heart disease; AF, atrial fibrillation.

^a^ Data are presented as number (%) or mean ± standard deviation or median and interquartile range.

^b^ Diabetes was defined as self-reported physician diagnosis of diabetes or FPG concentration ≥7.0 mmol/L or use of Hypoglycemic drugs.

**Figure 2:**
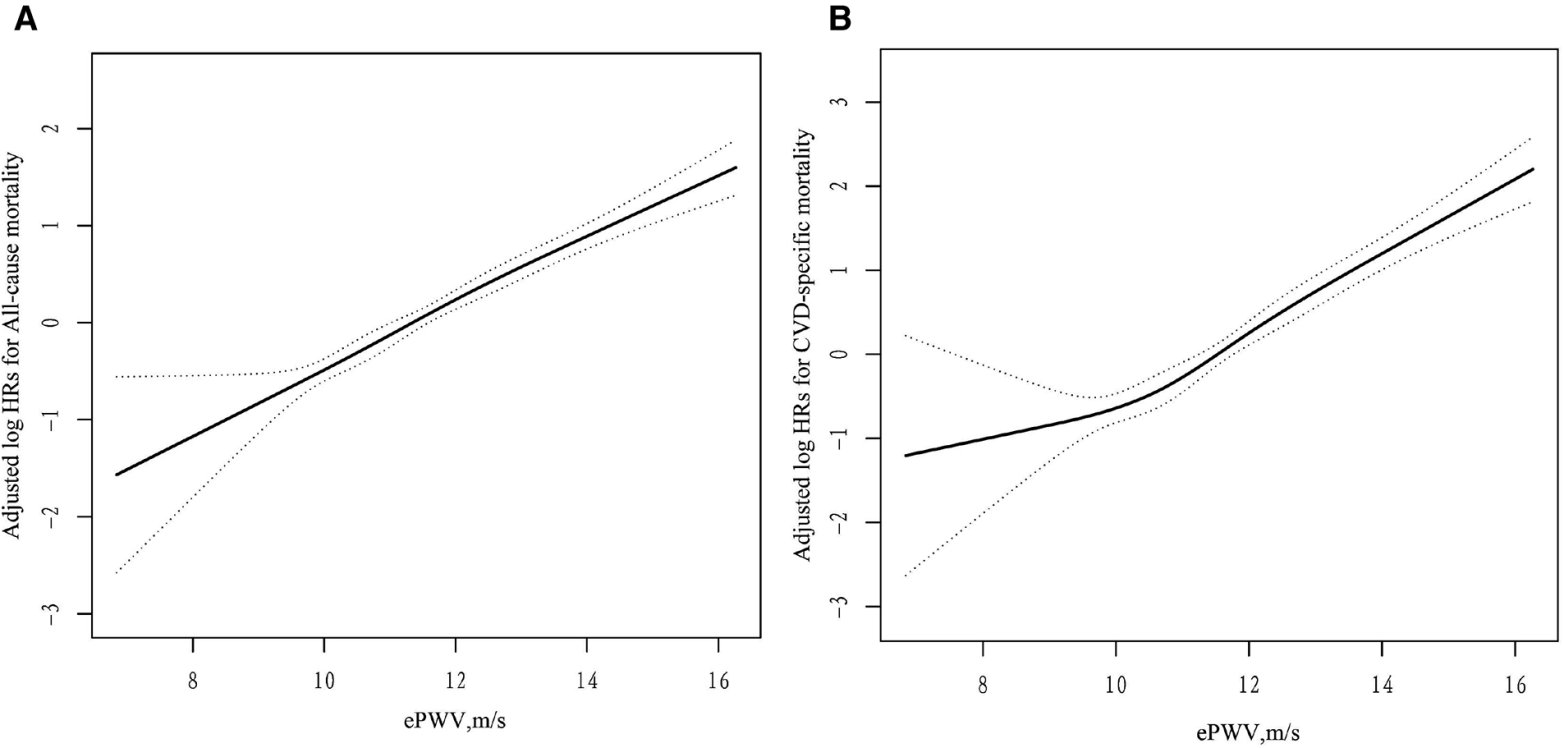
Dose-response relationships between ePWV and the risk of all-cause (**A**) and CVD-specifc* (**B**) mortality. Adjustment factors included sex, BMI, heart rate, diabetes,stroke, CHD, AF, smoking status, drinking status, Hcy, FPG, TC, TG, uric acid, HDL-c, LDL-c, serum albumin, eGFR, antihypertensive drugs, hypoglycemic drugs, lipid-lowering drugs, antiplatelet drugs. *cardiovascular mortality.

**Figure 3:**
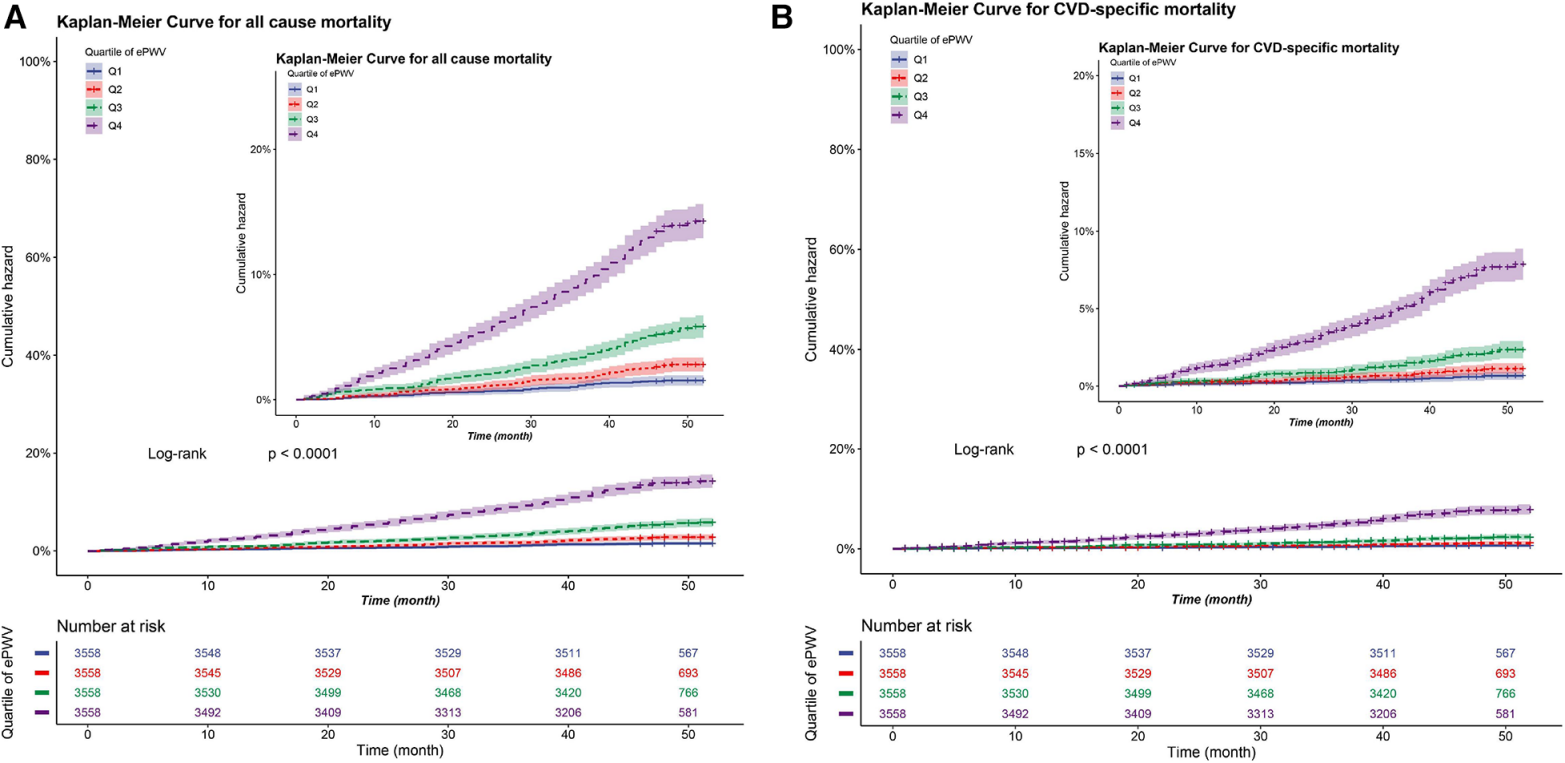
Cumulative incidence of all-cause and CVD-specifc mortality in patients with hypertensive according to quartiles of ePWV (cm/s). (**A**) Cumulative incidence curve for all-cause mortality. (**B**) Cumulative incidence curve for CVD-specifc mortality*. *cardiovascular mortality.

Cox proportional hazards regression analysis was used to construct three regression models for covariate adjustment. The results are presented in Table 2. In the fully adjusted model (model 3), we observed a significant association between each 1 m/s increase in ePWV and 37% (HR: 1.37, 95% CI: 1.31–1.43) and 52% (HR: 1.52, 95% CI: 1.43–1.62) elevated risks all-cause and cardiovascular mortalities, respectively. According to ePWV quartile, compared with Q1 (ePWV < 10.25), the HRs for all-cause mortality in Q2 (10.25 < ePWV < 11.32), Q3 (11.32 < ePWV < 12.40), and Q4 (ePWV ≥ 12.40) groups were 1.50 (HR: 1.50, 95% CI: 1.07–2.10), 2.34 (HR: 2.34, 95% CI: 1.73–3.18), and 4.09 (HR: 4.09, 95% CI: 3.05–5.49), respectively. The HR (95% CI) for cardiovascular mortality in the Q2, Q3, and Q4 groups were 1.54 (0.92, 2.58), 2.42 (1.51, 3.87), and 5.93 (3.80, 9.24) respectively (all *P* for trend <0.001). The dose-response relationship between ePWV, cardiovascular mortality, and all-cause mortality is depicted in Figure 2, indicating a positive correlation between ePWV and mortality events. The Kaplan–Meier survival curve demonstrated a sequential increase in cumulative cardiovascular mortality and all-cause mortality among patients in ePWV Q1, Q2, Q3, and Q4 (Figure 3).

**Table 2:** Hazard ratios and 95% confidence intervals of all cause mortality and cardiovascular mortality.

ePWV, m/s	Events (%)	H*R* (95% CI), *P* value		
		Model 1	Model 2	Model 3
All-cause mortality				
Per 1 m/s increase	806 (5.66%)	1.66 (1.60, 1.73) <0.0001	1.55 (1.49, 1.61)<0.0001	1.37 (1.31, 1.43)<0.0001
Q1(<10.25)	54 (1.52%)	1	1	1
Q2 (10.25<11.32)	98 (2.75%)	1.82 (1.31, 2.54) 0.0004	1.74 (1.25, 2.42) 0.001	1.50 (1.07, 2.10) 0.017
Q3 (11.32–<12.40)	191 (5.37%)	3.59 (2.65, 4.86) <0.0001	3.16 (2.33, 4.28)<0.0001	2.34 (1.73, 3.18)<0.0001
Q4(≥12.40)	463 (13.01%)	9.09 (6.86, 12.05) <0.0001	7.03 (5.29, 9.36)<0.0001	4.09 (3.05, 5.48)<0.0001
*P* for trend		<0.0001	<0.0001	<0.0001
Cardiovascular mortality				
per 1 m/s increase	397 (2.79%)	1.78 (1.69, 1.88)<0.0001	1.68 (1.59, 1.78)<0.0001	1.52 (1.43, 1.62)<0.0001
Q1(<10.25)	23 (0.65%)	1	1	1
Q2 (10.25–<11.32)	40 (1.12%)	1.75 (1.05, 2.92) 0.033	1.68 (1.01, 2.81) 0.047	1.54 (0.92, 2.58) 0.100
Q3 (11.32–<12.40)	78 (2.19%)	3.45 (2.16, 5.49)<0.0001	3.10 (1.94, 4.94)<0.0001	2.42 (1.51, 3.87) 0.0002
Q4 (≥12.40)	256 (7.20%)	11.81 (7.71, 18.10) <0.0001	9.47 (6.15, 14.57) <0.0001	5.93 (3.80, 9.24)<0.0001
*P* for trend		<0.0001	<0.0001	<0.0001

Model 1 was adjusted for none.

Model 2 was adjusted for sex, BMI, heart rate.

Model 3 was adjusted for sex, BMI, heart rate, diabetes,stroke, CHD, AF, smoking status, drinking status, Hcy, FPG, TC, TG, uric acid, HDL-c, LDL-c, serum albumin, eGFR, antihypertensive drugs, hypoglycemic drugs, lipid-lowering drugs, antiplatelet drugs.

**Figure 4:**
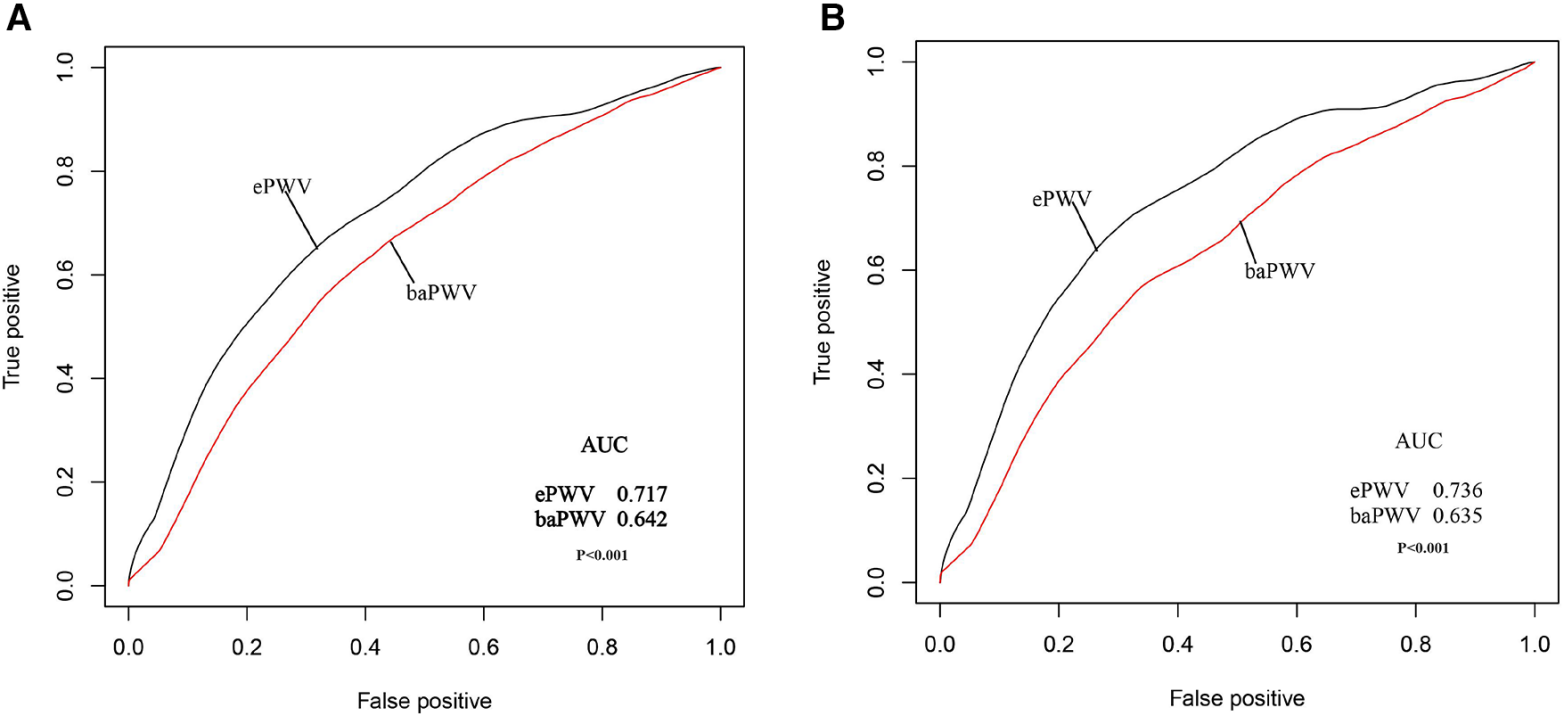
Receiver operating characteristic (ROC) curves for prediction of all-cause (**A**) and CVD-specifc mortality*. (**B**) A comparison of the ePWV with the baPWV. *cardiovascular mortality.

The AUC values used to compare the predictive abilities of ePWV and baPWV for all-cause and cardiovascular mortalities demonstrated the superior predictive ability of the ePWV for both all-cause (AUC: 0.717 vs. 0.642) and cardiovascular (AUC: 0.736 vs. 0.635) mortalities compared with baPWV (Figure 4). The ability to predict all-cause and cardiovascular mortality was further assessed by comparing the NRI and IDI between the two indices. In predicting all-cause mortality, ePWV demonstrated a 29.8% increase in NRI and a 3.2% increase in IDI compared with baPWV. Similarly, the ePWV exhibited a comparable increase in predicting cardiovascular mortality (Table 3).

**Table 3:** Comparison of the predictive ability of ePWV and baPWV for all-cause mortality and cardiovascular mortality.

ePWV VS. baPWV	Effect value	95% CI	*P* value
All-cause mortality			
NRI	0.298	0.212-0.363	<0.0001
IDI	0.032	0.015-0.048	<0.0001
Cardiovascular mortality			
NRI	0.385	0.278-0.470	0.033
IDI	0.022	0.001-0.045	<0.0001

The results of subgroup analyses of the association of ePWV with all-cause and cardiovascular mortalities stratified by sex, age, BMI, current smoking, current drinking, antihypertensive drugs, hypoglycemic drugs, SBP, and DBP are shown in Supplementary Figure S1. The interactions of ePWV with BMI and hypoglycemic drugs were statistically significant (*P* for interaction = 0.020 and 0.028, respectively). A higher ePWV was associated with higher all-cause mortality in participants with BMI ≥ 25 kg/m^2^ and with higher cardiovascular mortality in participants not taking hypoglycemic drugs. Except for BMI and the use of hypoglycemic drugs, no other stratified variables showed an interaction with ePWV. A series of sensitivity analyses was also conducted on diverse populations. Supplementary Table S1 shows the results of the analyses after excluding patients who died within one year. Supplementary Table S2 shows the results of the analyses after excluding patients with ABI ≤ 0.9. Finally, Supplementary Table S3 shows the results of the analyses after excluding patients who died of cancer. Cox proportional hazards regression models were used to assess the associations among ePWV, cardiovascular mortality, and all-cause mortality in the three cohorts. These findings demonstrate a consistent relationship between increased ePWV and elevated mortality risk.

### Discussion

Our analysis of data from a longitudinal cohort of patients with hypertension in China revealed a significant association between ePWV and cardiovascular and all-cause mortalities. As ePWV increased, the risks of cardiovascular and all-cause mortalities gradually increased. This relationship was independent of traditional cardiovascular risk factors. The results of the subgroup analyses demonstrated heterogeneous results attributed to varying outcomes, in which BMI significantly modified the positive correlation between ePWV and all-cause mortality. Additionally, high ePWV was associated with a significant increase in the risk of cardiovascular mortality among patients who did not receive hypoglycemic drugs. Finally, our findings indicated that ePWV outperformed baPWV in predicting both cardiovascular and all-cause mortalities.

Since its introduction by Greve et al. in 2016, the ePWV has attracted significant attention among researchers who have investigated its correlations with cardiovascular disease and all-cause mortality and compared it to conventional risk factors. Ji et al. conducted a prospective cohort study involving 98,348 individuals undergoing physical examinations in the Kailuan cohort to assess the association between ePWV and cardiovascular events as well as all-cause mortality (mean EPWV: 9.11 ± 2.01 m/s). These findings demonstrated the significant relationship between ePWV and cardiovascular disease and all-cause mortality risks even after adjusting for traditional cardiovascular risk factors (22). Moreover, Liu et al. utilized data from the China Longitudinal Study on Health and Retirement (CHARLS), comprising 13,116 older adult individuals in China, to evaluate the impact of baseline and dynamic ePWV levels during follow-up. Following a median follow-up period of seven years, the findings revealed that elevated baseline ePWV and increasing ePWV levels during follow-up were associated with an increased risk of all-cause mortality in this population (21). Hsu et al. (23) investigated the relationship among ePWV, baPWV, and cardiovascular mortality in a cohort of 881 individuals undergoing physical examinations in Taiwan. Assessment of the comparative predictive abilities of these measures for mortality revealed that both ePWV and baPWV demonstrated significant prognostic value for cardiovascular mortality; however, ePWV exhibited superior predictive ability. Moreover, this previous study assessed these predictive capacities for cardiovascular mortality only in individuals without pre-existing conditions. Thus, the outcomes may not be universally applicable because of the limited sample size, with potential variations in sensitivity across different disease states. Hsu et al. conducted a prospective cohort study to assess the impact of ePWV on cardiovascular and all-cause mortalities in 187 patients with myocardial infarction. The findings revealed that, following a median follow-up period of 73 months, ePWV demonstrated an independent predictive ability for cardiovascular mortality after accounting for other risk factors. However, its predictive ability for all-cause mortality was observed only in univariate regression analysis (24).

In their prospective cohort study of 33,930 participants from the National Nutrition Cohort Study, Cheng et al. observed that ePWV was an independent risk factor for all-cause and cardiovascular mortalities among American adults and remained unaffected by traditional cardiovascular risk factors following a median follow-up period of 133 months. Moreover, this index exhibited a robust predictive value for both all-cause and cardiovascular mortalities, surpassing the performance of the Framingham Risk Score (Framingham) and Combined Cohort Equation (PCE) (32). Greve et al. demonstrated that the ePWV exhibited a higher predictive value in untreated than in treated patients with hypertension (16).The secondary analysis of the SPRINT study further demonstrated that ePWV exhibited a robust predictive value in patients with hypertension and effectively reflected the impact of intensive blood pressure management on arterial stiffness. Among 8,450 patients with hypertension with a median follow-up duration of 3.2 years in the SPRINT study, Charalambos et al. (17) demonstrated that the ePWV surpassed the Framingham Risk Score in accurately predicting cardiovascular events, thereby suggesting its supplementation to enhance the accuracy of cardiovascular disease risk prediction. Moreover, the group receiving intensive blood pressure therapy showed a notable decrease in the ePWV progression rate, leading to a relative reduction in cardiovascular event occurrence. Prospective cohort studies conducted across diverse racial populations have consistently demonstrated that the ePWV can serve as an independent prognostic indicator for overall mortality (18–20). Combined, these findings underscore the significance of ePWV as an autonomous predictor of cardiovascular events and mortality risk.

We validated our research hypotheses through a prospective study of the Wuyuan hypertension cohort, thereby expanding the research population and broadening the applicability of the ePWV index. Additionally, we provided two novel insights. First, we demonstrated that the ePWV outperformed the baPWV in predicting cardiovascular and all-cause mortalities among patients with hypertension. Specifically, the ePWV exhibited superior prognostic capability for assessing the risk of all-cause and cardiovascular deaths in individuals with hypertension. The comparison of these measures contributes to understanding the feasibility of utilizing PWV as a substitute index without requiring specialized equipment. Furthermore, we observed heterogeneity in mortality outcomes among various subgroups. In particular, patients with ePWV and BMI ≥ 25 kg/m^2^ exhibited a more pronounced risk of all-cause mortality. This could be attributed to their increased susceptibility to metabolic disorders and the confounding effects of cardiovascular disease risk factors. Epidemiological studies have consistently demonstrated a significant association between overweight and obesity and an elevated all-cause mortality risk (33, 34). Therefore, it is imperative to emphasize weight loss and BMI reduction among patients with hypertension who are also overweight or obese to mitigate the risk of mortality associated with increased ePWV. Furthermore, the association between ePWV and cardiovascular mortality was more pronounced among individuals not taking hypoglycemic medications. This suggests that ePWV holds substantial significance as an independent risk indicator of cardiovascular mortality in this population. Furthermore, hypoglycemic agents can mitigate cardiovascular mortality risk by ameliorating glucose metabolism disorders. Metabolic disturbances or impaired vascular insulin sensitivity may alter arterial wall function and structure, leading to increased arterial stiffness (35). Therefore, the adverse effects of ePWV on cardiovascular mortality in patients taking hypoglycemic drugs were partially mitigated. However, previous studies have not reported such interactions; thus, the impact of hypoglycemic drugs on arterial stiffness progression and patient prognosis requires further study. These findings underscore the significance of the ePWV in predicting overall mortality, as well as disease-specific and cardiovascular mortalities, particularly among individuals who are overweight or obese and those not taking hypoglycemic medications. The assessment of ePWV can provide clinicians with crucial insights into the risk of cardiovascular and all-cause mortalities, aiding in the early identification and intervention of high-risk individuals to enhance patient prognosis and health outcomes.

While the biological mechanisms underlying the association between ePWV and cardiovascular and all-cause mortality remain unclear, we propose several possible explanations. First, the ePWV is a calculated index using age and average blood pressure, which provides an accurate reflection of the severity of vascular aging. Vascular aging involves multiple factors, including oxidative stress, chronic inflammation, and cellular senescence (36). Cardiovascular and non-cardiovascular diseases that lead to related deaths follow the same pathological process (37). An elevated ePWV may serve as an indicator of arterial stiffness and vascular dysfunction, which reflect abnormalities in vascular function. As a hallmark of aging, arterial stiffness is a crucial manifestation of vascular senescence (38). The development of arterial stiffness is characterized by the deposition of collagen and calcium, or the degradation of elastin induced by hemodynamics, leading to alterations in arterial wall thickness and function, as well as changes in elastin content. These modifications impact vascular tension and compliance, thereby emerging as important determinants of multi-organ injury (39, 40). These pathological mechanisms are intricately linked to the overall health status of patients and their susceptibility to cardiovascular diseases. Furthermore, an increase in arterial stiffness results in a heightened exposure of target organs to pulsatile hemodynamics, potentially leading to barotrauma-induced injury. This phenomenon is a potential intermediary factor contributing to increased mortality risk (41).

The strengths of the present cohort study include the large-scale population of patients with hypertension in China, prospective design, extended duration, high follow-up rate, reliable and comprehensive questionnaire survey, and standardized physical examination. These factors significantly enhanced the accuracy of the ePWV calculations. This investigation holds substantial public health significance for the management of patients with primary hypertension, particularly in rural regions of China, where the prevalence of hypertension is generally high and awareness and treatment rates remain relatively low. Moreover, given the unfavorable prognosis among patients in these areas, with limited resources available for the comprehensive management of hypertension, our findings demonstrate that a precise estimation of ePWV can be achieved by considering age and blood pressure levels alone. The effective monitoring of ePWV in patients with hypertension and timely interventions can help mitigate cardiovascular events and enhance the prognosis of hypertension, thereby demonstrating its robust clinical applicability. Recent research also highlights that enhancing comprehensive nursing care for patients with hypertension in low- and middle-income countries and regions can significantly alleviate the burden of cardiovascular diseases and ameliorate the impact of socioeconomic inequality (42). The findings of this study have significant implications for the development of region-specific health strategies under resource constraints, thereby contributing to improving patients' overall well-being.

When interpreting these results, the following limitations must be acknowledged. First, only baseline ePWV values were obtained, whereas multiple measurements of ePWV can provide more precise data on arterial stiffness. Secondly, the effects of heart rate on blood pressure and pulse wave velocity should also be considered. We accounted for this by adjusting for heart rate, thereby mitigating any associated effects. Third, owing to the progressive nature of arteriosclerosis, PWV cannot predict short-term cardiovascular risk factors; however, it holds significant potential for estimating arterial stiffness in the medium and long term. Therefore, ePWV-based estimation of arterial stiffness demonstrates considerable long-term value. As this study included only patients with hypertension, further validation of these findings across a diverse population is required.

### Conclusions

In conclusion, ePWV was superior to baPWV as an independent predictor of cardiovascular and all-cause mortalities. Higher ePWV significantly increases the risk of all-cause mortality in overweight and obese patients, and the relationship between higher ePWV levels and increased risk of cardiovascular death is more significant in patients who do not take hypoglycemic drugs. In fact, considering the management and prevention of hypertension in rural areas of China, it is more necessary to find indicators as easily available as ePWV to predict cardiovascular events and deaths in high-risk patients with hypertension. This has important public health significance for chronic disease management in primary medical care.

## Data Availability

The raw data supporting the conclusions of this article will be made available by the authors upon reasonable request. Requests to access the datasets should be directed to XC, xiaoshumenfan126@163.com.

## References

[1] Roth GA Mensah GA Johnson CO Addolorato G Ammirati E Baddour LM Global burden of cardiovascular diseases and risk factors, 1990–2019: update from the GBD 2019 study. J Am Coll Cardiol. (2020) 76(25):2982–3021. 10.1016/j.jacc.2020.11.010 33309175 PMC7755038

[2] Roth GA Forouzanfar MH Moran AE Barber R Nguyen G Feigin VL Demographic and epidemiologic drivers of global cardiovascular mortality. N Engl J Med. (2015) 372(14):1333–41. 10.1056/NEJMoa1406656 25830423 PMC4482354

[3] Camici GG Liberale L. Aging: the next cardiovascular disease. Eur Heart J. (2017) 38(21):1621–3. 10.1093/eurheartj/ehx239 29447349

[4] Ni YQ Zhan JK Liu YS. Roles and mechanisms of MFG-E8 in vascular aging-related diseases. Ageing Res Rev. (2020) 64:101176. 10.1016/j.arr.2020.101176 32971257

[5] Duprez DA De Buyzere MM De Bruyne L Clement DL Cohn JN. Small and large artery elasticity indices in peripheral arterial occlusive disease (PAOD). Vasc Med. (2001) 6(4):211–4. 10.1177/1358836(0100600402 11958385

[6] Boutouyrie P Chowienczyk P Humphrey JD Mitchell GF. Arterial stiffness and cardiovascular risk in hypertension. Circ Res. (2021) 128(7):864–86. 10.1161/CIRCRESAHA.121.318061 33793325

[7] Niiranen TJ Kalesan B Larson MG Hamburg NM Benjamin EJ Mitchell GF Aortic-brachial arterial stiffness gradient and cardiovascular risk in the community: the framingham heart study. Hypertension. (2017) 69(6):1022–8. 10.1161/HYPERTENSIONAHA.116.08917 28396534 PMC5426958

[8] Chen Y Shen F Liu J Yang GY. Arterial stiffness and stroke: de-stiffening strategy, a therapeutic target for stroke. Stroke Vasc Neurol. (2017) 2(2):65–72. 10.1136/svn-2016-000045 28959494 PMC5600012

[9] Blacher J Pannier B Guerin AP Marchais SJ Safar ME London GM. Carotid arterial stiffness as a predictor of cardiovascular and all-cause mortality in end-stage renal disease. Hypertension. (1998) 32(3):570–4. 10.1161/01.hyp.32.3.570 9740628

[10] Mansia G De Backer G Dominiczak A Cifkova R Fagard R Germano G 2007 ESH-ESC guidelines for the management of arterial hypertension: the task force for the management of arterial hypertension of the European society of hypertension (ESH) and of the European society of cardiology (ESC). Blood Press. (2007) 16(3):135–232. 10.1080/08037050701461084 17846925

[11] Mitchell GF Parise H Benjamin EJ Larson MG Keyes MJ Vita JA Changes in arterial stiffness and wave reflection with advancing age in healthy men and women: the framingham heart study. Hypertension. (2004) 43(6):1239–45. 10.1161/01.HYP.0000128420.01881.aa 15123572

[12] Choi JC Lee JS Kang SY Kang JH Bae JM Lee DH. Limitation of brachial-ankle pulse wave velocity in assessing the risk of stroke: importance of instantaneous blood pressure. Cerebrovasc Dis. (2009) 27(5):417–25. 10.1159/000209236 19295204

[13] Umemura S Arima H Arima S Asayama K Dohi Y Hirooka Y The Japanese society of hypertension guidelines for the management of hypertension (JSH 2019). Hypertens Res. (2019) 42(9):1235–481. 10.1038/s41440-019-0284-9 31375757

[14] Jang SY Ju EY Huh EH Kim JH Kim DK. Determinants of brachial-ankle pulse wave velocity and carotid-femoral pulse wave velocity in healthy Koreans. J Korean Med Sci. (2014) 29(6):798–804. 10.3346/jkms.2014.29.6.798 24932081 PMC4055813

[15] Townsend RR Wilkinson IB Schiffrin EL Avolio AP Chirinos JA Cockcroft JR Recommendations for improving and standardizing vascular research on arterial stiffness: a scientific statement from the American heart association. Hypertension. (2015) 66(3):698–722. 10.1161/HYP.0000000000000033 26160955 PMC4587661

[16] Greve SV Blicher MK Kruger R Sehestedt T Gram-Kampmann E Rasmussen S Estimated carotid-femoral pulse wave velocity has similar predictive value as measured carotid-femoral pulse wave velocity. J Hypertens. (2016) 34(7):1279–89. 10.1097/HJH.0000000000000935 27088638

[17] Vlachopoulos C Terentes-Printzios D Laurent S Nilsson PM Protogerou AD Aznaouridis K Association of estimated pulse wave velocity with survival: a secondary analysis of SPRINT. JAMA Netw Open. (2019) 2(10):e1912831. 10.1001/jamanetworkopen.2019.12831 31596491 PMC6802234

[18] Laugesen E Olesen K Peters CD Buus NH Maeng M Botker HE Estimated pulse wave velocity is associated with all-cause mortality during 8.5 years follow-up in patients undergoing elective coronary angiography. J Am Heart Assoc. (2022) 11(10):e025173. 10.1161/JAHA.121.025173 35535599 PMC9238554

[19] Heffernan KS Jae SY Loprinzi PD. Association between estimated pulse wave velocity and mortality in U.S. adults. J Am Coll Cardiol. (2020) 75(15):1862–4. 10.1016/j.jacc.2020.02.035 32299599

[20] Heffernan KS Wilmoth JM London AS. Estimated pulse wave velocity and all-cause mortality: findings from the health and retirement study. Innov Aging. (2022) 6(7):igac056. 10.1093/geroni/igac056 36284701 PMC9585457

[21] Liu HR Li CY Xia X Chen SF Lu XF Gu DF Association of estimated pulse wave velocity and the dynamic changes in estimated pulse wave velocity with all-cause mortality among middle-aged and elderly Chinese. Biomed Environ Sci. (2022) 35(11):1001–11. 10.3967/bes2022.129 36443253

[22] Ji C Gao J Huang Z Chen S Wang G Wu S Estimated pulse wave velocity and cardiovascular events in Chinese. Int J Cardiol Hypertens. (2020) 7:100063. 10.1016/j.ijchy.2020.100063 33447784 PMC7803041

[23] Hsu PC Lee WH Tsai WC Chen YC Chu CY Yen HW Comparison between estimated and brachial-ankle pulse wave velocity for cardiovascular and overall mortality prediction. J Clin Hypertens (Greenwich). (2021) 23(1):106–13. 10.1111/jch.14124 33314741 PMC8030022

[24] Hsu PC Lee WH Tsai WC Chi NY Chang CT Chiu CA Usefulness of estimated pulse wave velocity in prediction of cardiovascular mortality in patients with acute myocardial infarction. Am J Med Sci. (2021) 361(4):479–84. 10.1016/j.amjms.2020.10.023 33637306

[25] Li M Zhan A Huang X Hu L Zhou W Wang T Positive association between triglyceride glucose index and arterial stiffness in hypertensive patients: the China H-type hypertension registry study. Cardiovasc Diabetol. (2020) 19:139. 10.1186/s12933-020-01124-2 32948181 PMC7501677

[26] Levey AS Stevens LA Schmid CH Zhang YL Castro AF 3rd Feldman HI A new equation to estimate glomerular filtration rate. Ann Intern Med. (2009) 150:604–12. 10.7326/0003-4819-150-9-200905050-00006 19414839 PMC2763564

[27] Giral P Neumann A Weill A Coste J. Cardiovascular effect of discontinuing statins for primary prevention at the age of 75 years: a nationwide population-based cohort study in France. Eur Heart J. (2019) 40(43):3516–25. 10.1093/eurheartj/ehz458 31362307 PMC6855142

[28] Li Y Qin X Luo L Wang B Huo Y Hou FF Folic acid therapy reduces the risk of mortality associated with heavy proteinuria among hypertensive patients. J Hypertens. (2017) 35(6):1302–9. 10.1097/HJH.0000000000001292 28441699

[29] Determinants of pulse wave velocity in healthy people and in the presence of cardiovascular risk factors: “establishing normal and reference values”. Eur Heart J. (2010) 31(19):2338–50. 10.1093/eurheartj/ehq165 20530030 PMC2948201

[30] Schultz MG Picone DS Armstrong MK Black JA Dwyer N Roberts-Thomson P The influence of SBP amplification on the accuracy of form-factor-derived mean arterial pressure. J Hypertens. (2020) 38(6):1033–9. 10.1097/HJH.0000000000002385 32371792

[31] Papaioannou TG Protogerou AD Vrachatis D Konstantonis G Aissopou E Argyris A Mean arterial pressure values calculated using seven different methods and their associations with target organ deterioration in a single-center study of 1878 individuals. Hypertens Res. (2016) 39(9):640–7. 10.1038/hr.2016.41 27194570

[32] Cheng W Kong F Pan H Luan S Yang S Chen S. Superior predictive value of estimated pulse wave velocity for all-cause and cardiovascular disease mortality risk in U.S. general adults. BMC Public Health. (2024) 24(1):600. 10.1186/s12889-024-18071-2 38402165 PMC10893621

[33] Bhaskaran K Dos-Santos-Silva I Leon DA Douglas IJ Smeeth L. Association of BMI with overall and cause-specific mortality: a population-based cohort study of 3·6 million adults in the UK. Lancet Diabetes Endocrinol. (2018) 6(12):944–53. 10.1016/S2213-8587(18)30288-2 30389323 PMC6249991

[34] Liu XM Liu YJ Zhan J He QQ. Overweight, obesity and risk of all-cause and cardiovascular mortality in patients with type 2 diabetes mellitus: a dose-response meta-analysis of prospective cohort studies. Eur J Epidemiol. (2015) 30(1):35–45. 10.1007/s10654-014-9973-5 25421785

[35] Henry RM Kostense PJ Spijkerman AM Dekker JM Nijpels G Heine RJ Arterial stiffness increases with deteriorating glucose tolerance status: the hoorn study. Circulation. (2003) 107(16):2089–95. 10.1161/01.CIR.0000065222.34933.FC 12695300

[36] Maloberti A Vallerio P Triglione N Occhi L Panzeri F Bassi I Vascular aging and disease of the large vessels: role of inflammation. High Blood Press Cardiovasc Prev. (2019) 26(3):175–82. 10.1007/s40292-019-00318-4 31054064

[37] Ungvari Z Tarantini S Donato AJ Galvan V Csiszar A. Mechanisms of vascular aging. Circ Res. (2018) 123(7):849–67. 10.1161/CIRCRESAHA.118.311378 30355080 PMC6248882

[38] Shirwany NA Zou MH. Arterial stiffness: a brief review. Acta Pharmacol Sin. (2010) 31(10):1267–76. 10.1038/aps.2010.123 20802505 PMC3078647

[39] Mitchell GF. Arterial stiffness in aging: does it have a place in clinical practice?: recent advances in hypertension. Hypertension. (2021) 77(3):768–80. 10.1161/HYPERTENSIONAHA.120.14515 33517682

[40] Zieman SJ Melenovsky V Kass DA. Mechanisms, pathophysiology, and therapy of arterial stiffness. Arterioscler Thromb Vasc Biol. (2005) 25(5):932–43. 10.1161/01.ATV.0000160548.78317.29 15731494

[41] Chirinos JA Segers P Hughes T Townsend R. Large-artery stiffness in health and disease: JACC state-of-the-art review. J Am Coll Cardiol. (2019) 74(9):1237–63. 10.1016/j.jacc.2019.07.012 31466622 PMC6719727

[42] Stein DT Reitsma MB Geldsetzer P Agoudavi K Aryal KK Bahendeka S Hypertension care cascades and reducing inequities in cardiovascular disease in low- and middle-income countries. Nat Med. (2024) 30(2):414–23. 10.1038/s41591-023-02769-8 38278990

