# Supplementary material for: Estimated pulse wave velocity as a predictor of all-cause and cardiovascular mortality in patients with hypertension in China: a prospective cohort study
Source: Front Cardiovasc Med. 2024 Apr 29;11:1365344. doi: 10.3389/fcvm.2024.1365344 (PMC11089216; doi:10.3389/fcvm.2024.1365344)
Supplement: Supplementary file 1 [file Datasheet1.docx]

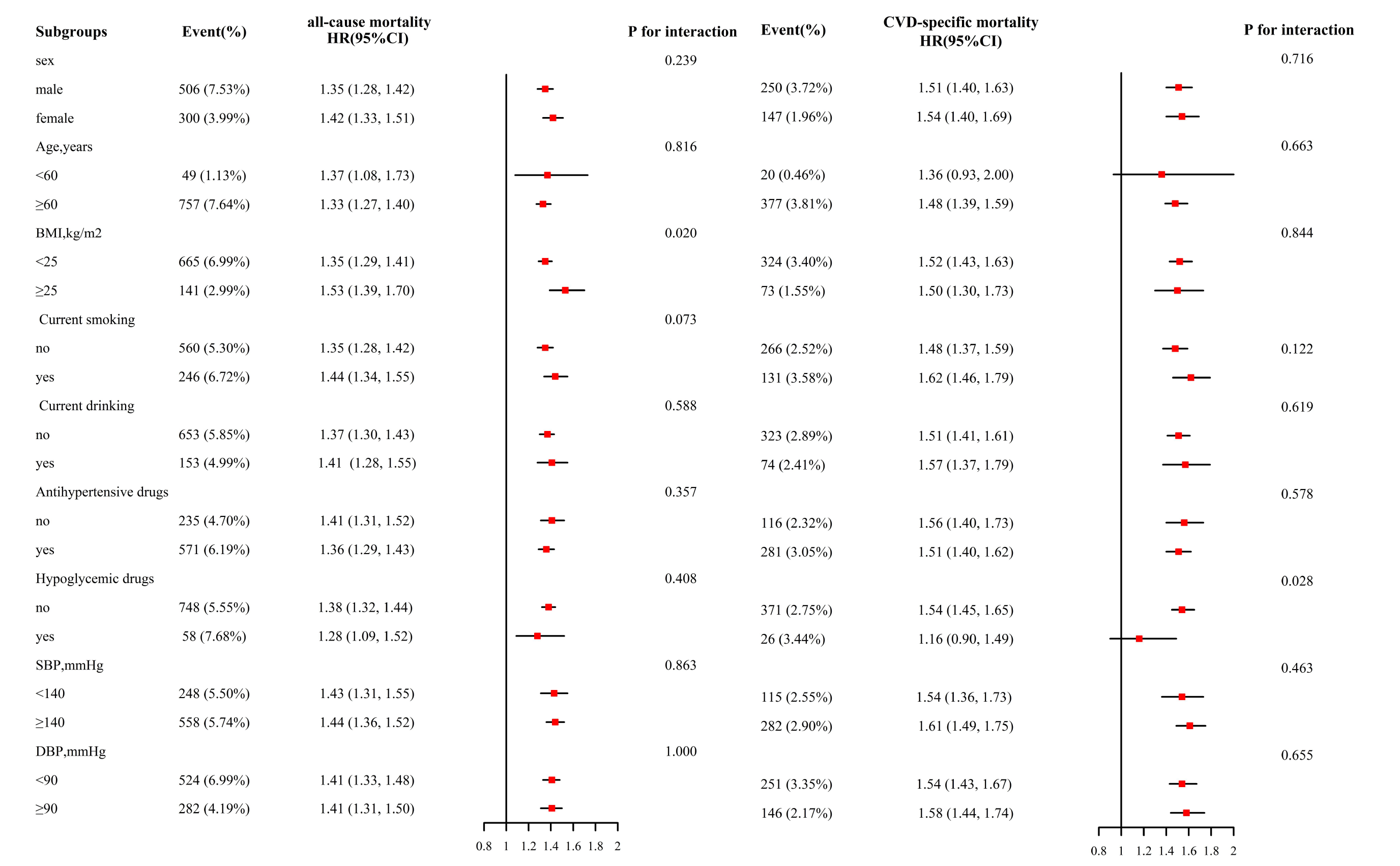


**Supplementary Figure 1 Association between ePWV and the risk of all-cause and CVD-specifc mortality in various subgroups.** Each subgroup analysis adjusted, if not stratified, sex, BMI, pulse, diabetes,stroke, CHD, AF, smoking status, drinking status, Hcy, FBG, TC, TG, uric acid, HDL-c, LDL-c, serum albumin, eGFR, antihypertensive drugs, hypoglycemic drugs, lipid-lowering drugs, antiplatelet drugs.

Supplementary Table 1 Hazard ratios and 95% confidence intervals of allcause mortality and CVD-specific mortality (excluding patients who died within 1 year)

| ePWV, m/s | Events (%) | HR (95%CI), P value | |  |
| --- | --- | --- | --- | --- |
|  |  | Model 1 | Model 2 | Model 3 |
| All-cause mortality |  |  |  |  |
| per 1 m/s increase | 657 (4.67%) | 1.67 (1.60, 1.75) <0.0001 | 1.56 (1.50, 1.64) <0.0001 | 1.40 (1.33, 1.47) <0.0001 |
| Q1(<10.25) | 43 (1.21%) | 1 | 1 | 1 |
| Q2 (10.25 <11.32) | 81 (2.29%) | 1.96 (1.36, 2.83) 0.0003 | 1.86 (1.29, 2.69) 0.0009 | 1.60 (1.10, 2.32) 0.013 |
| Q3 (11.32 to<12.40) | 158 (4.48%) | 3.74 (2.67, 5.24) <0.0001 | 3.27 (2.33, 4.59) <0.0001 | 2.44 (1.74, 3.44) <0.0001 |
| Q4(≥12.40) | 375 (10.81%) | 9.52 (6.94, 13.04) <0.0001 | 7.34 (5.33, 10.09) <0.0001 | 4.40 (3.17, 6.11) <0.0001 |
| *P for trend* |  | <0.0001 | <0.0001 | <0.0001 |
| CVD-specific mortality |  |  |  |  |
| per 1 m/s increase | 322 (2.29%) | 1.82 (1.71, 1.93) <0.0001 | 1.72 (1.61, 1.83) <0.0001 | 1.56 (1.45, 1.67) <0.0001 |
| Q1(<10.25) | 18 (0.51%) | 1 | 1 | 1 |
| Q2 (10.25 to<11.32) | 30 (0.85%) | 1.73 (0.97, 3.09) 0.0642 | 1.65 (0.92, 2.95) 0.0919 | 1.49 (0.83, 2.68) 0.177 |
| Q3 (11.32 to<12.40) | 66 (1.87%) | 3.74 (2.22, 6.29) <0.0001 | 3.30 (1.95, 5.56) <0.0001 | 2.56 (1.51, 4.33) 0.0005 |
| Q4 (≥12.40) | 208 (5.99%) | 12.58 (7.77, 20.36) <0.0001 | 9.86 (6.06, 16.03) <0.0001 | 6.25 (3.80, 10.30) <0.0001 |
| *P for trend* |  | <0.0001 | <0.0001 | <0.0001 |

Non-adjusted model adjust for: None

Adjust I model adjust for: sex, BMI, pulse

Adjust II model adjust for: sex, BMI, pulse, diabetes,stroke, CHD, AF, smoking status, drinking status, Hcy, FBG, TC, TG, uric acid, HDL-c, LDL-c, serum albumin, eGFR, antihypertensive drugs, hypoglycemic drugs, lipid-lowering drugs, antiplatelet drugs

Supplementary Table 2 Hazard ratios and 95% confidence intervals of allcause mortality and CVD-specific mortality (excluding patients with ABI≤0.9)

| ePWV, m/s | Events (%) | HR (95%CI), P value | |  |
| --- | --- | --- | --- | --- |
|  |  | Model 1 | Model 2 | Model 3 |
| All-cause mortality |  |  |  |  |
| per 1 m/s increase | 520 (4.93%) | 1.64 (1.56, 1.72) <0.0001 | 1.54 (1.46, 1.62) <0.0001 | 1.37 (1.29, 1.44) <0.0001 |
| Q1(<10.25) | 40 (1.53%) | 1 | 1 | 1 |
| Q2 (10.25 <11.32) | 63 (2.31%) | 1.52 (1.02, 2.25) 0.0396 | 1.44 (0.97, 2.14) 0.0710 | 1.19 (0.80, 1.78) 0.3833 |
| Q3 (11.32 to<12.40) | 137 (5.10%) | 3.38 (2.38, 4.81) <0.0001 | 2.97 (2.08, 4.23) <0.0001 | 2.15 (1.50, 3.08) <0.0001 |
| Q4(≥12.40) | 280 (11.07%) | 7.58 (5.44, 10.55) <0.0001 | 5.92 (4.23, 8.28) <0.0001 | 3.49 (2.47, 4.95) <0.0001 |
| P for trend |  | <0.0001 | <0.0001 | <0.0001 |
| CVD-specific mortality |  |  |  |  |
| per 1 m/s increase | 247 (2.34%) | 1.79 (1.67, 1.92) <0.0001 | 1.70 (1.58, 1.83) <0.0001 | 1.55 (1.43, 1.68) <0.0001 |
| Q1(<10.25) | 17 (0.65%) | 1 | 1 | 1 |
| Q2 (10.25 to<11.32) | 20 (0.73%) | 1.13 (0.59, 2.16) 0.7061 | 1.09 (0.57, 2.08) 0.7992 | 0.95 (0.49, 1.81) 0.8651 |
| Q3 (11.32 to<12.40) | 57 (2.12%) | 3.31 (1.93, 5.69) <0.0001 | 2.97 (1.73, 5.12) <0.0001 | 2.27 (1.31, 3.94) 0.0034 |
| Q4 (≥12.40) | 153 (6.05%) | 9.75 (5.91, 16.10) <0.0001 | 7.91 (4.76, 13.13) <0.0001 | 5.08 (3.00, 8.58) <0.0001 |
| P for trend |  | <0.0001 | <0.0001 | <0.0001 |

Non-adjusted model adjust for: None

Adjust I model adjust for: sex, BMI, pulse

Adjust II model adjust for: sex, BMI, pulse, diabetes,stroke, CHD, AF, smoking status, drinking status, Hcy, FBG, TC, TG, uric acid, HDL-c, LDL-c, serum albumin, eGFR, antihypertensive drugs, hypoglycemic drugs, lipid-lowering drugs, antiplatelet drugs

Supplementary Table 3 Hazard ratios and 95% confidence intervals of allcause mortality and CVD-specific mortality (excluding patients who died from cancer)

| ePWV, m/s | Events (%) | HR (95%CI), P value | |  |
| --- | --- | --- | --- | --- |
|  |  | Model 1 | Model 2 | Model 3 |
| All-cause mortality |  |  |  |  |
| per 1 m/s increase | 626 (4.45%) | 1.74 (1.67, 1.82) <0.0001 | 1.62 (1.55, 1.69) <0.0001 | 1.41 (1.34, 1.48) <0.0001 |
| Q1(<10.25) | 41 (1.16%) | 1 | 1 | 1 |
| Q2 (10.25 <11.32) | 65 (1.84%) | 1.60 (1.08, 2.36) 0.019 | 1.52 (1.03, 2.24) 0.037 | 1.30 (0.88, 1.93) 0.193 |
| Q3 (11.32 to<12.40) | 134 (3.83%) | 3.35 (2.36, 4.75) <0.0001 | 2.92 (2.06, 4.15) <0.0001 | 2.07 (1.45, 2.95) <0.0001 |
| Q4(≥12.40) | 386 (11.09%) | 10.08 (7.31, 13.91) <0.0001 | 7.66 (5.53, 10.61) <0.0001 | 4.06 (2.91, 5.68) <0.0001 |
| P for trend |  | <0.0001 | <0.0001 | <0.0001 |
| CVD-specific mortality |  |  |  |  |
| per 1 m/s increase | 397 (2.83%) | 1.79 (1.69, 1.89) <0.0001 | 1.69 (1.59, 1.79) <0.0001 | 1.52 (1.42, 1.62) <0.0001 |
| Q1(<10.25) | 23 (0.65%) | 1 | 1 | 1 |
| Q2 (10.25 to<11.32) | 40 (1.13%) | 1.75 (1.05, 2.93) 0.0321 | 1.69 (1.01, 2.82) 0.046 | 1.57 (0.94, 2.63) 0.086 |
| Q3 (11.32 to<12.40) | 78 (2.23%) | 3.47 (2.18, 5.53) <0.0001 | 3.12 (1.96, 4.97) <0.0001 | 2.45 (1.53, 3.92) 0.0002 |
| Q4 (≥12.40) | 256 (7.35%) | 11.92 (7.78, 18.27) <0.0001 | 9.55 (6.20, 14.69) <0.0001 | 5.96 (3.82, 9.29) <0.0001 |
| P for trend |  | <0.0001 | <0.0001 | <0.0001 |

Non-adjusted model adjust for: None

Adjust I model adjust for: sex, BMI, pulse

Adjust II model adjust for: sex, BMI, pulse, diabetes,stroke, CHD, AF, smoking status, drinking status, Hcy, FBG, TC, TG, uric acid, HDL-c, LDL-c, serum albumin, eGFR, antihypertensive drugs, hypoglycemic drugs, lipid-lowering drugs, antiplatelet drugs
